# Preventing HIV Transmission in Chinese Internal Migrants: A Behavioral Approach

**DOI:** 10.1155/2014/319629

**Published:** 2014-12-25

**Authors:** Xiaona Liu, Vicki Erasmus, Xinying Sun, Rui Cai, Yuhui Shi, Jan Hendrik Richardus

**Affiliations:** ^1^Department of Public Health, Erasmus MC, University Medical Center Rotterdam, P.O. Box 2040, 3000 CA Rotterdam, Netherlands; ^2^Department of Social Medicine and Health Education, School of Public Health, Peking University, Beijing 100191, China

## Abstract

This study is a step towards a behavioral intervention to prevent HIV transmission among Chinese internal migrants. To explore important and changeable determinants of condom use and inspect effective and feasible methods to increase condom use for the target population, we conducted a three-round web-based Delphi study among a panel of 62 experts between October 2012 and March 2013. The panelists were purposely selected using a stepwise procedure to represent topic-related areas of expertise. The response rate per round ranges from 21% to 81%. The panelists identified 19 possible determinants of condom use and reported 16 intervention methods they considered successful. They agreed that attitude towards condom use was the most important and changeable determinant, while applying behavioral theory, increasing sexual education and condom access, performing worksite health promotion, detecting risk factors, and working closely with relevant organizations and the government were effective and feasible methods to increase condom use among internal migrants in China. In conclusion, results of this study highlight the importance of attitude in changing condom use and underscore the need to apply behavior theory and integrate multiple educational approaches for developing behavioral HIV prevention interventions targeting internal migrants in China.

## 1. Introduction

Chinese internal migrants, also known as the “floating population,” are of substantial importance to preventing HIV transmission in the country [1]. Since the economic reform of the late 1970s, internal mobility has increased significantly as workers, driven by large social and economic disparities, try to find better employment opportunities, higher income, and a more attractive lifestyle in cities [2]. According to the National Bureau of Statistics, there were 145.3 million Chinese internal migrants in 2009 [3]. One study of the national surveillance data estimated that 0.075% of them were living with an HIV infection in 2009 and projected that the prevalence of HIV among the internal migrants will reach 0.11% in 2015 [4]. The internal migrants have an important impact on the HIV epidemic in China for the following two reasons: (1) high mobility, because they frequently shift between jobs and locations and pay seasonal visits back home, which may further spread HIV infection to others and (2) frequent engagement in unsafe sexual behaviors, such as commercial sex and unprotected sex [5].

Condom use has been identified as one of the most efficient means available to reduce the risk of HIV transmission [6]. It was estimated that consistent condom users are 10 to 20 times less likely to acquire HIV compared with inconsistent ones or nonusers [7]. Despite the attention paid in recent years to the importance of condom use by the government and organizations dedicated to the fight against HIV/AIDS, condom use by internal migrants in both committed and casual relationships remains low. A cross-sectional survey of 1625 Chinese migrant construction laborers, the major component of the internal migrants, revealed that most participants had never used condoms during sex with their stable partners; 14.2% of participants indicated having had sex with a sex worker, but only 35.4% of them reported consistent condom use when having commercial sex [8]. In the absence of a guideline on HIV prevention at both regional and national levels, behavioral changing programs—especially programs aiming at increasing condom use—remain central to efforts aimed at decreasing the risk of sexual transmission of HIV among the internal migrants in China.

This study is an essential step in the development of a behavioral HIV prevention intervention among Chinese internal migrants following the protocol of Intervention Mapping (IM). As one of the systematic approaches to planning health promotion programs, IM maps the path from recognition of a need or problem to the identification of a solution [9]. Selecting important and changeable determinants of the health behavioral outcome is one of its core processes used to understand the problem and problem-causing factors. Possible determinants include personal determinants (such as cognitive factors and capabilities) and environmental conditions (such as social influences and structural influences). Previous studies have identified many possible determinants of condom use among Chinese internal migrants, such as gender, risk perception, and self-efficacy [10]. However, little is known about the performance of those determinants in terms of their importance (i.e., strength of association with the behavior) and changeability (i.e., how likely it is that the intervention is going to influence a change in the determinant). On the other hand, selecting effective and feasible intervention methods, another of IM's core processes, ensures the practical application of the developed program. To date, a number of studies have described behavioral and psychosocial interventions that aim to increase condom use by Chinese internal migrants [11], but many of these are obscure about the methods used and lack evidence regarding the performance of those methods in terms of effectiveness (i.e., how much it changes the behavior) and feasibility (i.e., how likely it is that the method would be practical in a forthcoming intervention).

This study aims to explore possible determinants of condom use and intervention methods that aim to increase condom use among Chinese internal migrants, as well as to assess the performance of identified determinants and intervention methods. Given the available wide range of topic-relevant colleagues who have profound knowledge and experience on design and implementation HIV prevention interventions worldwide, adopting the Delphi technique is an optimal route to achieve our goal. The Delphi technique allows us to apply an iterative process to obtain previously unknown opinions, that is, HIV interventions among internal migrants in China, and builds consensus in a group of informed individuals. Also, this technique is one of the best-known methods for dealing with the open-ended and creative aspects of a problem because it motivates independent thought and gradual formation of group solutions [12, 13]. By using Delphi method, we are able to collect a combination of qualitative and quantitative information, which will be of extensive interest to researchers, health educators, and policy makers who wish to understand the sexual behavior of Chinese internal migrants and make practical contributions to promote HIV prevention among them.

## 2. Methods

### 2.1. Study Design

We conducted a three-round Delphi study with panelists who were provided with web-based questionnaires between October 2012 and March 2013. In the first round the panelists proposed possible determinants of condom use and past and/or ongoing intervention methods aimed at increasing condom use among Chinese internal migrants. In the following two rounds they judged the performance of each determinant and intervention method. The procedure of this study is characterized by iteration, anonymity (the panelists were not aware of each other's participation), and feedback (subsequent rounds were held using both the information introduced in the previous round and the statistical summaries of group results). We developed and hosted the web survey internally using secure university domains of LimeSurvey, an open source survey application [14].

### 2.2. Participants

We purposively selected the panelists using a five-step procedure recommended by Okoli and Pawlowski [12]. First, the five areas of possible expertise involved were identified (i.e., epidemiology; psychology; social and behavioral science; health education and promotion; health policy and management). Then each area was populated with the names of individuals, as well as their email address, research filed, nationality, and work site. This information was derived from several sources, including publications on topic, previous research participation, professional email lists, and advisory panel involvement. After that, we created a list for each stakeholder area and ranked experts based upon their relevance with the studied topic. Finally, we invited 62 experts, who evenly represented the five identified areas of expertise, and were knowledgeable about HIV prevention in China. All panel members were contacted directly via an email, which included a unique URL link to the survey, and asked to nominate other experts if possible. Invitation letters and questionnaires for all rounds contained an introduction with basic information on the studied population, that is, Chinese internal migrants, who are defined in this study as Chinese citizens living in an area different from their household registration (the “hukou” system) within China.

### 2.3. Questionnaire

The first questionnaire consisted of four open questions serving as the source of creative input for idea generation. Based on their knowledge and experience, the panelists were first asked to list the following: (1) possible determinants of condom use among Chinese internal migrants in general; (2) determinants specifically influencing condom use during sex with a committed sex partner (in this study this included sex within marriage, engagement, courtship, civil union, and close long-term relationships); (3) determinants specifically influencing condom use during sex with a casual sex partner (in this study this includes short-term extramarital sex, commercial sex, one-time encounters, and sex in the absence of emotional attachment or love); and (4) past and/or ongoing intervention methods aimed at increasing condom use among Chinese internal migrants. The answers in the first round were then organized and they provided the content of the following questionnaires.

In the second and third questionnaires the determinants and intervention methods identified in the first questionnaire were presented for rating in six domains: (1) the importance of the identified determinants of condom use among Chinese internal migrants in general; (2) the changeability of the identified factors regarding condom use in general; (3) the importance of the identified determinants in committed sexual relationships; (4) the importance of the identified determinants in casual sexual relationships; (5) the effectiveness of the identified intervention methods at increasing condom use among Chinese internal migrants; and (6) the feasibility of the identified intervention methods. The panelists were asked to score each determinant and intervention method on a 5-point Likert scale with the following ratings: 1 = Very  low; 2 = Low; 3 = Neutral; 4 = High; 5 = Very  high. In the second and third questionnaires a response was compulsory for each question to ensure a complete data set [15].

All questionnaires and invitation letters were provided in both Chinese and English. The panelists could read and answer the surveys in either of the two languages. Each survey was issued formally after a pilot survey was conducted among six researchers (three Chinese and three Dutch), who were asked to provide feedback and comments about the statements, process, instructions, and barriers with regard to completing the survey. On average, the first questionnaire could be completed in less than 25 minutes, the second one took about 20 minutes, and the last one took about 15 minutes.

### 2.4. Data Analysis

The qualitative answers provided in the first round were reviewed and structured into the subsequent questionnaires by two authors, who were proficient in both languages. The data in the second and third round were analyzed using SPSS version 20.0. The median and interquartile deviation (IQD) were calculated as statistical measures for all ordinal variables. The median represents the 50th percentile value of opinions, and the IQD represents the distance between the 25th and 75th value of opinions. As a rule, it was decided that panelists have reached consensus on an item if that item had an IQD ≤ 1. If consensus was reached on an item in round two, it was removed from the third questionnaire. Thus, all items with an IQD > 1 in the second round were reported and maintained in the third questionnaire.

## 3. Results

### 3.1. Study Population

In the first round survey we invited 62 panelists from China (36), the United States (15), Netherlands (8), and Belgium (3) to participate, of whom 13 (21%) completed the questionnaire (Table 1). Two people declined to continue participating due to lack of time, and four additional people, who were nominated by the participating panelists, were invited to participate in the second round. Of the 64 panelists invited to participate in the second round survey, 16 (25%) completed the questionnaire, and of those, 13 (81%) completed the third-round survey. Overall, 21% of our original sample participated in all three rounds of the study (*n* = 13). Among the five identified areas of expertise, the panelists with expertise in health education and promotion had the highest response rate (31%). Panelists who answered in Chinese had a similar response rate as panelists who answered in English (21% and 22%, resp.).

### 3.2. Determinants of Condom Use among Chinese Internal Migrants

The panelists identified 19 determinants of condom use in general among Chinese internal migrants (Table 2), 6 additional determinants for committed sexual relationships (25 in total), and 8 additional determinants for casual sexual relationships (27 in total). The identified determinants covered both personal factors (i.e., knowledge, attitudes, risk perception, self-efficacy, and skills) and socioenvironmental conditions (i.e., social norms and environmental support). They reached consensus (IQD ≤ 1) for all determinants with regard to their importance and changeability for condom use in Chinese internal migrants (*n* = 27). Almost all of the identified determinants were judged as being important (median ≥ 4) and changeable (median ≥ 4), except for the following two determinants which were considered to be less important (median = 3): embarrassment of picking up free condoms and access to sexual health services.

The following two determinants were rated to be the most important (median = 5) and to have the highest level of changeability (median = 4): the attitude of Chinese internal migrants towards using condoms and the attitude of sexual partners of Chinese internal migrants towards using condoms. Moreover, the former was an especially important determinant of condom use in casual sexual relationships (mean = 4.5), while the latter was especially important in committed sexual relationships (mean = 5). The following three determinants were rated as having high importance (median = 4) but low changeability: financial reasons (median = 3), peer pressure (median = 3.5), and social support (median = 3.5). In addition to the identified general determinants of condom use, the taboo nature of premarital sex was identified as an important social influence on condom use in committed sexual relationships, while structural determinants (i.e., barriers to using condoms due to violence, loss of clients and payment, sex venue, and the use of alternative means of contraception) were identified as playing important structural influences in casual sexual relationships.

### 3.3. Intervention Methods Aimed at Increasing Condom Use among Chinese Internal Migrants

The panelists identified 16 past and/or ongoing intervention methods aimed at increasing condom use among Chinese internal migrants, covering 4 methods of intervention development, 10 intervention approaches, and 2 process measures (Table 3). They reached consensus (IQD ≤ 1) for 14 methods (88%) on their effectiveness at increasing condom use and for 15 methods (94%) on their feasibility. All identified intervention methods were judged as being effective (median ≥ 4) and feasible (median ≥ 4).

The panelists agreed that using behavioral theories is the most effective (median = 5) and feasible (median = 4) method for developing successful interventions. They also agreed that evaluating interventions is important (median = 5) but had varied opinions on the feasibility of evaluations (median = 4, IQD = 1.5). Regarding intervention approaches, half of the identified approaches were about training and education, while others involved condom access, worksite health promotion, and detection of risk factors. Furthermore, they agreed that improving health education on safe sex behavior is a very feasible approach to improving condom use (median = 5). However, they agreed less on the effectiveness of this approach (median = 4, IQD = 1.5). For process measures, they all agreed that cooperating with the government and relevant organizations was not only an effective (median = 4) method but also a feasible (median = 4) method.

## 4. Discussion

The panelists in this study identified 19 possible determinants of condom use among Chinese internal migrants in general ranging from personal cognitive factors and skills to environmental conditions. They also reported 16 past/ongoing intervention methods aimed at increasing condom use among the population that they considered successful, including methods of intervention development, intervention approaches, and process measures. The panelists reached consensus on the fact that attitude towards condom use is the most important and changeable determinant. They also agreed that applying behavioral theory, conducting training and education, increasing condom access, performing worksite health promotion, detecting risk factors, and working closely with relevant organizations and the government are effective and feasible methods to increase condom use among internal migrants in China.

This is the first study that uses both the qualitative and quantitative data from a panel of international experts to understand condom use in general and provide applicable insight into increasing condom use among Chinese internal migrants in particular. This is also the first time Intervention Mapping approach has been applied in a study involving Chinese internal migrants. The results were obtained from panelists who have a variety of expertise and come from around the world, and yet they reached a strong consensus on almost all of the questions. Several limitations, however, need to be considered when interpreting the findings. This study reached a general low response rate over three rounds that may introduce certain response bias. It is, however, worth noting that the response rate is comparable to that of other studies using similar data collecting method [16, 17]. A strength of the study is that the panelists had a great deal of valuable expertise in the area of behavioral HIV prevention interventions (including many key experts in this field). More of the panelists with expertise in health policy and management dropped out than the panelists with other expertise, which could have influenced the results. Finally, internal migrants constitute many diverse groups with varied socioeconomic status living under different environmental conditions in China. The unique data collected in this study can be broadly applied as a first step in intervention development and can be integrated with results from specific field efforts, such as observations, field surveys, and face-to-face interviews for specific target groups.

The determinants and intervention methods identified through the Delphi technique provide practical input for the design of future interventions aimed at increasing condom use among Chinese internal migrants. First, all panelists thought that, due to their personal economic situation, Chinese internal migrants perhaps cannot afford to buy condoms and that this is an important but less changeable factor influencing their condom use. At the same time, the panelists considered that distributing free condoms to the population is both highly effective and feasible way to improve condom use. This underscores the need for future interventions that aim to increase condom use among the population to include free condom distribution. Second, half of the identified past and/or ongoing HIV prevention intervention approaches are about training or education, which is in line with the findings of a review of relevant intervention programs [18]. The panelists agreed that knowledge is an important and changeable determinant of condom use and acknowledged that health education on safe sex behavior is also feasible. However, they agreed less on whether or not increasing knowledge would increase actual condom use among the Chinese internal migrants. If it would not increase actual condom use, programs focusing only on increasing the level of knowledge regarding HIV/STDs and sexual behavior among Chinese internal migrants are insufficient to actually change risk behavior. Our study identified many other cognitive factors and personal skills associated with condom use, which were also found in other studies on condom use, such as lack of negotiation skills [19], self-efficacy [20], desire for trust [21], and personal perception about sex partners [22]. In accordance with the fact that the finding of our previous review on condom promotion intervention conducted in the past decade suggests a theory-based intervention was more likely to be successful [23], the panelists noted that following one of the behavioral theories (e.g., theory of planned behavior [24]) is crucial, regarding both effectiveness and feasibility, for developing a successful HIV prevention intervention. The core constructs of behavioral theories have a great potential to inform intervention planners about the possible determinants of condom use and to help them reinforce multiple determinants in a structured manner.

The results of this study suggest that attitude is strongly associated with condom use and should therefore be targeted in future interventions. Previous research studies have also highlighted the importance of attitude, as one of the social-cognitive factors, for both explaining and predicting condom use [25]. The theory of planned behavior suggests that attitude is one of the factors causing behavioral intention and is composed of both a positive or negative evaluation of a particular behavior and belief about the outcome of the behavior [26]. It would therefore seem that efforts (using identified intervention approaches such as training, culture-tailored worksite health promotion, and adopting new media) need to be designed to sharpen positive attitudes about condom use. The efforts should aim to show that using condoms would make life more productive and would be beneficial to the health of internal migrants in China. Finally, future interventions should pay special attention to environmental conditions. The panelists in this study identified that social influences, including peer pressure and social support, are important for condom use in general, which is in line with the findings of other studies [27]. Regarding the behavior in different sexual relationships, social influences were identified as important determinants in committed sexual relationships, while structural influences were identified as important determinants in casual sexual relationships. This finding, together with the suggestion from the panelists, stresses the need for greater cooperation with relevant organizations and the government in order to increase condom use. For example, gaining commitment and support at the administrative level may be beneficial for improving the unfavorable social influences and organizational climate facing internal migrants in China.

In conclusion, the results of this Delphi study are critical input for systematically developing an HIV prevention intervention that aims to increase condom use among internal migrants in China. For an intervention targeting this population to be successful, messages should be built upon a behavioral theory integrating cognitive factors, personal skills, and environmental conditions. Moreover, the interventions should focus on shaping positive attitudes towards condom use, applying multiple intervention approaches (e.g., free condom distribution, training, and worksite health promotion), and realizing commitment and support from the government and relevant organizations.

## Figures and Tables

**Table 1:** Description of the panelists by Delphi round.

	Round 1	Round 2	Round 3	Overall response (%)
Area of expertise				
Epidemiology	1/12	3/12	3/3	3/12 (25%)^*^
Psychology	3/12	3/13	2/3	2/12 (17%)
Social and behavioral science	3/13	3/13	3/3	3/13 (23%)
Health education and promotion	3/13	4/14	4/4	4/13 (31%)
Health policy and management	3/12	3/12	1/3	1/12 (8%)
Applied language				
Chinese	10/39	10/39	8/10	8/39 (21%)
English	3/23	6/25	5/6	5/23 (22%)
Nationality				
Chinese	10/36	11/36	8/11	8/36 (22%)^*^
American	1/15	2/15	2/2	2/15 (13%)
European (Dutch; Belgian)	2/11	3/13	3/3	3/11 (27%)
Overall response (%)	13/62 (21%)	16/64 (25%)	13/16 (81%)	13/62 (21%)

^*^ *P *< 0.05.

**Table 2:** Sociopsychological and environmental determinants of condom use and their performances of importance and changeability among Chinese internal migrants.

Determinants	Importance		Changeability		Determinants	Importance		Changeability	
Personal cognition and capability	Median	IQD	Median	IQD	Environmental condition	Median	IQD	Median	IQD
Socioeconomic status					Social norm				
(1) Financial reasons	4	1	3	0	(15) Peer pressure	4	1	3.5	1
Knowledge					(16) Role models	4	1	4	1
(2) Knowledge of HIV/STD	4	1	4	1	(17) Social support	4	1	3.5	1
(3) Knowledge of safe sex behavior	4	1	4	1	Environmental feasibility				
(4) Knowledge of condom use	4	1	4	1	(18) Inconvenience to carry condoms	4	1	4	1
Attitudes					(19) Access to sexual health services	3	1	4	1
(5) Attitude from sex partner	5	1	4	1					
(6) Own attitude towards condom use	5	1	4	1					
(7) Beliefs on ease of use of condoms	4	1	4	1					
(8) Use of condom feeling during sex	4	1	4	1					
(9) Risk perception	4	1	4	1					
(10)Extent of familiarity	4	1	4	0.5					
Self-efficacy									
(11) Confidence on the use of condom	4	1	4	1					
(12) Embarrassed to pick up free condoms	3	1	4	1					
(13) Unaware that free condoms are available	4	1	4	0.75					
Skills									
(14) Open communication with partner	4	0.5	4	1					

Note: *N*_*d*_: number of determinants; IQD: interquartile deviation; the panelists score in a 5-point Likert scale, 1 = Very low; 2 = Low; 3 = Neutral; 4 = High; 5 = Very high.

**Table 3:** Intervention methods aiming at increasing condom use and their performance of effectiveness and feasibility among Chinese internal migrants.

Methods	Effectiveness		Feasibility	
	Median	IQD	Median	IQD
Intervention development				
(1) Use of behavioral theories for the development of interventions	5	1	4	1
(2) Implement participatory approach	4	1	4	0.75
(3) Adapt interventions to the results of monitoring	4	1	4	1
(4) Effect and process evaluation during interventions	5	1	4	1.5
Approaches				
Training and education				
(5) Health education of knowledge on safe sex behavior	4	1.5	5	1
(6) Peer education	4	1	4	1
(7) Training on skills of negotiation and decision marking	4	1	4	1
(8) Improve health literacy	4	0	4	0
(9) Implement various forms of education	4	1	4	1
Condom access				
(10) Distribution of free condoms	4	1	4	0.5
(11) Make condoms widely available in their life environments	4	1	4	1
Worksite health promotion				
(12) Culture-tailored health promotion in their worksites	4	1	4	1
Detection risk factors				
(13) Detection of risk factors for HIV transmission	4	1.5	4	0
(14) Apply new media in an intervention	4	0.75	4	0.75
Process measures				
(15) Make relevant organizations commit and cooperate in every level	4	0.5	4	1
(16) Support from the government	4	1	4	0

Note: IQD: interquartile deviation; the panelists score in a 5-point Likert scale, 1 = Very low; 2 = Low; 3 = Neutral; 4 = High; 5 = Very high.
